# How to make a red flower: the combinatorial effect of pigments

**DOI:** 10.1093/aobpla/plw013

**Published:** 2016-03-01

**Authors:** Julienne Ng, Stacey D. Smith

**Affiliations:** Department of Ecology and Evolutionary Biology, University of Colorado, Boulder, CO 80309, USA

**Keywords:** Anthocyanins, biochemical pathway, carotenoids, flower colour, pigments, Solanaceae

## Abstract

Red flowers have evolved repeatedly across angiosperms and are frequently examined in an ecological context. However, less is known about the biochemical basis of red colouration in different taxa. In this study, we examine the spectral properties, anthocyanin composition and carotenoid expression of red flowers in the tomato family, Solanaceae, which have evolved independently multiple times across the group. Our study demonstrates that Solanaceae typically make red flowers either by the sole production of red anthocyanins or, more commonly, by the dual production of purple or blue anthocyanins and orange carotenoids. In using carotenoids to modify the effect of purple and/or blue anthocyanins, these Solanaceae species have converged on the same floral hue as those solely producing red anthocyanins, even when considering the visual system of pollinators. The use of blue anthocyanins in red flowers appears to differ from other groups, and suggests that the genetic changes underlying evolutionary shifts to red flowers may not be as predictable as previously suggested.

## Abstract (precis)

This study examines the biochemical basis of red flowers in the tomato family, Solanaceae. We show that red-flowered species have converged on the same floral hue using either the sole production of red anthocyanin pigments or, more commonly, the dual production of purple or blue anthocyanins and orange carotenoid pigments. The use of blue anthocyanins in red flowers appears to differ from other groups, and suggests that the genetic changes underlying evolutionary transitions to red flowers may not be as predictable as previously suggested.

## Introduction

Flower colour plays a central role in plant ecology, not only mediating signalling to pollinators (Bradshaw and Schemske 2003; Fenster *et al*. 2004) but also contributing to thermoregulation (Anderson *et al*. 2013) and defence against herbivores (Strauss *et al*. 2004). In terms of its genetic basis and biochemistry, pigmentation is one of the most well-characterized plant traits (Winkel-Shirley 2001; Tanaka *et al*. 2008). Three main classes of pigments contribute to flower colour: anthocyanins, carotenoids and betalains. While betalains are only found in Carophyllales (Brockington *et al*. 2015), anthocyanins and carotenoids are widespread among angiosperms (Grotewold 2006). Anthocyanins are water-soluble flavonoid compounds that are stored in plant vacuoles, and contribute to red, pink, blue and purple colours. In contrast, carotenoids are hydrophobic yellow, orange and red compounds that are produced and stored in plastids. These two common plant pigments are known to occur both alone and in combination with floral tissue (e.g. van Raamsdonk 1993; Schemske and Bradshaw 1999; Irwin and Strauss 2005), although species whose flowers are solely coloured by carotenoids appear to be relatively rare (Ohmiya 2011).

From an evolutionary perspective, changes in flower pigmentation are among the most common transitions in angiosperm traits, and have led to the wide diversity of flower colour across the angiosperm phylogeny (Rausher 2008; Smith and Goldberg 2015). Changes in the concentration of pigments present in the flower can alter the intensity of colouration, while shifts in pigment composition (i.e. the presence or absence of different anthocyanins, carotenoids or betalains) can alter the floral hue (Tanaka *et al*. 2008; Streisfeld and Rausher 2009*b*; Yuan *et al*. 2013). Additional factors such as cellular pH and co-pigments can modify the colours of these pigments and thus also affect floral hue (Forkmann 1991; Mol *et al*. 1998; Whitney and Glover 2007). At a macroevolutionary scale, frequent shifts in flower colour have resulted in widespread patterns of convergent evolution, with similar colours evolving multiple times independently, even within the same family or genus (Perret *et al*. 2003; Wilson *et al*. 2007; Tripp and Manos 2008).

**Figure 1.**
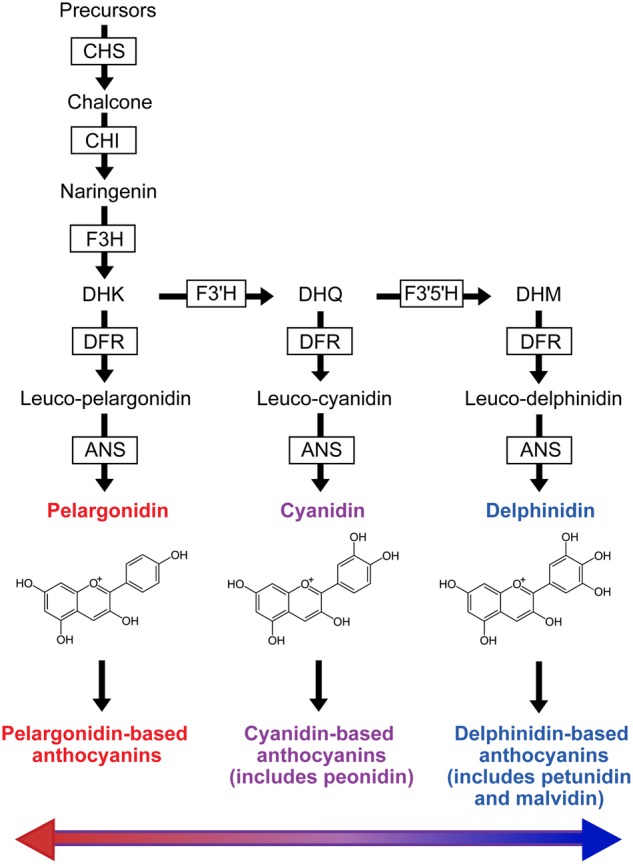
Simplified version of the flavonoid biosynthetic pathway showing the production of the three major types of anthocyanin pigments (the glycosides of anthocyanidins): those derived from pelargonidin (mainly red), from cyanidin (mainly purple) and from delphinidin (mainly blue). These are distinguished by their level of hydroxylation: one B-ring hydroxyl group for pelargonidin, two for cyanidin, three for delphinidin. Arrows indicate enzymatic reactions with enzymes shown in boxes, and products and precursors outside of boxes. ANS, anthocyanidin synthase; CHI, chalcone isomerase; CHS, chalcone synthase; DFR, dihydroflavonol-4-reductase; DHK, dihydrokaempferol; DHM, dihydromyricetin; DHQ, dihydroquercetin; F3H, flavanone 3-hydroxylase; F3′H, flavonoid 3′-hydroxylase; F3′5′H, flavonoid 3′,5′-hydroxylase.

Here, we focus on the biochemistry of red flowers, which have evolved repeatedly in angiosperms and are found in at least 65 families (Cronk and Ojeda 2008). Although red flower colouration has been frequently examined in an ecological context (e.g. Grant 1966; Campbell *et al*. 1997; Rodríguez-Gironés and Santamaría 2004), less is known about the biochemical basis of red colouration in different taxa. In some lineages, red flowers have evolved through the production of ‘red’ pelargonidin-based anthocyanins (Fig. 1) (e.g. Smith and Rausher 2011; Wessinger and Rausher 2015). In other species, red flowers are produced by the joint expression of anthocyanins and yellow–orange carotenoids. For example, red-coloured *Mimulus* flowers are due to the combination of carotenoids and mono- and di-hydroxylated anthocyanins derived from pelargonidin and cyanidin, respectively (Bradshaw and Schemske 2003; Streisfeld and Rausher 2009*a*). Overall, these findings suggest that different classes of pigments (e.g. carotenoids and anthocyanins) and/or different types of anthocyanins can be combined in various ways to make the same floral hue. However, the relative importance of carotenoid production, anthocyanin composition or other mechanisms for generating red colouration in flowers across different taxa remains unclear.

This study uses the tomato family, Solanaceae, to examine the range of anthocyanins in red flowers and the types of anthocyanins that co-occur with carotenoid pigments in these taxa. Recent phylogenetic studies indicate that red flowers have independently evolved at least 30 times in the family, and these origins are clustered within the last 10 million years of the family's roughly 50 million year history (Särkinen *et al*. 2013; Ng and Smith 2016). Both anthocyanin and carotenoid pigments are known to contribute to the diversity of flower colouration in Solanaceae (e.g. Beale *et al*. 1941; Ando *et al*. 1999; Ohmiya 2011), and indeed both classes of pigments have been documented in red-flowered species, with anthocyanins and the dual production of both anthocyanins and carotenoids most frequently used (Ng and Smith 2016). Here, we examine spectral properties, anthocyanin composition and carotenoid expression in 27 species that represent 24 of the estimated 30 independent origins of red colouration in Solanaceae to address three specific questions: (i) Does floral hue vary depending on the type of pigments present, i.e. do different pigment pathways lead to different shades of red? (ii) In red-flowered species that produce anthocyanins, what is the range of anthocyanin types in terms of hydroxylation? (iii) Does the type of anthocyanin depend on the presence or absence of carotenoids? We predict that yellow–orange carotenoids may commonly combine with blue and purple (cyanidin- and delphinidin-based) anthocyanins to result in an overall red floral hue. In contrast, red pelargonidin pigments alone may be sufficient to produce a red flower.

## Methods

### Sampling

We obtained floral tissue from 27 red-flowered Solanaceae species from the field, greenhouse-grown individuals and herbarium specimens **[see Supporting Information—Table S1]**. These species comprise representatives from all 12 genera in which red flowers independently evolved. As we were interested in the pigments involved in producing red flower colouration, we included species that are polymorphic for red colouration and those in which the corolla was partly red **[see Supporting Information—Table S1]**. For these species, we only conducted analyses using the red morph and the part of the corolla that was red, respectively.

### Characterizing anthocyanin types in red floral tissue

We used high-performance liquid chromatography (HPLC) to determine the range of anthocyanin types produced in red floral tissue across Solanaceae as well as their relative abundance. Anthocyanins are glycosylated forms of anthocyanidins, which may have one or more sugar moieties. Although there are many anthocyanins, most are derived from one of the six common anthocyanidins (pelargonidin, peonidin, cyanidin, petunidin, malvidin and delphinidin) (Gould *et al*. 2009). These six compounds fall into three major types based on their level of hydroxylation: (i) ‘red’ pelargonidin with one hydroxyl group; (ii) ‘purple’ cyanidin and its methylated derivative peonidin, which both have two hydroxyl groups; and (iii) ‘blue’ delphinidin and its methylated derivatives, petunidin and malvidin, which all have three hydroxyl groups (Fig. 1). We used HPLC to quantify each of the six anthocyanidins, and then grouped these into the three main anthocyanidin types (derived from pelargonidin, cyanidin or delphinidin; Fig. 1). For our analyses, we calculated the relative proportion of each anthocyanidin type found in the floral tissue of each species.

We conducted HPLC analyses using dried corolla tissue. For living collections, we collected corolla material in silica gel to desiccate. We used floral tissue from herbarium specimens for species from which we were unable to obtain fresh tissue **[see Supporting Information—Table S1]**. Floral herbarium tissue that remains coloured still has anthocyanins present (Ng and Smith 2016) and while the amount of anthocyanins is likely to degrade over time, our aim was to identify the type and relative proportion of anthocyanidins present, rather than to quantify the absolute amount. We extracted anthocyanidins from floral tissue following protocols outlined in Harborne (1984). We dried and re-eluted the extracts in 100 µL methanol with 1 % HCl. Following a protocol in A.E. Berardi, S. Hildreth, B.S.J. Winkel and S.D. Smith (in review), we analysed 50 µL with an Agilent Technologies 1260 Infinity liquid chromatography system with a photodiode array scanning from 200 to 600 nm at a step of 2 nm, and a 100 × 4.6 mm Chromolith Performance RP-18 endcapped column (EMD Chemicals, Darmstadt, Germany) at a flow rate of 1.1 mL min^−1^. We used solvent A (HPLC-grade water and 2 % trifluoroacetic acid, v/v) and solvent D (1-propanol and 0.1 % trifluoroacetic acid, v/v) to separate the anthocyanidins by gradient elution at 30 °C using the following programme: 0 % D from 0 to 8 min, increase to 7 % from 8 to 15 min, increase to 13 % from 15 to 18 min, increase to 15 % from 18 to 19 min, increase to 17 % from 19 to 20 min, increase to 27.5 % from 20 to 21 min, increase to 90 % from 21 to 27 min, increase to 95 % from 27 to 28 min and decrease to 0 % from 28 to 30 min. We used a standard solution of the six common anthocyanidins (Extrasynthese, Genay, France) to compare and identify the resulting peaks at 520 and 540 nm.

### Scoring the presence of carotenoids in floral tissue

Information on carotenoid presence in red-flowered taxa was taken from Ng and Smith (2016), who identified carotenoids from both fresh and herbarium tissue. We scored one additional species (*Nicotiana glauca*) using material sampled from the Steere herbarium (NY), as fresh floral tissue was unavailable **[see Supporting Information—Table S1]**. To identify the presence of floral carotenoids, we followed Ng and Smith's (2016) technique of examining petal cells using light microscopy. Carotenoids are produced and stored in chromoplasts and, therefore, appear in petal cells as discrete, coloured intracellular compartments (Tanaka *et al*. 2008). By examining herbarium tissue in both a dried and rehydrated state, we have previously shown that carotenoid presence can be reliably identified from herbarium specimens (Ng and Smith 2016).

### Comparing floral hue between different pigment pathways

We used phylogenetic comparative methods to test whether hue differed significantly depending on the pathway used to produce red colouration (i.e. only anthocyanins or dual production of anthocyanins and carotenoids). We computed measures of hue based on the spectral reflectance data from Ng and Smith (2016). These data are available for 22 of the 27 total species as reflectance can only be measured from fresh (not herbarium) tissue **[see Supporting Information—Table S1]**. First, we considered *λ*_Rmid_ (the reflectance at the midpoint between the minimum and maximum reflectance), which is a common metric that has been used to characterize colour in a range of taxa (e.g. Pryke *et al*. 2001; Hofmann *et al*. 2006; Cummings 2007; Ng *et al*. 2013). Red flowers should have higher reflectance in the red portion of the visible spectrum (650–700 nm) and thus high *λ*_Rmid_ values. Given that the hue of a flower may be perceived differently by different pollinators, we also computed two measures considering hummingbird and bee visual systems. Hummingbirds use four types of cones for colour perception (UV, short-, medium- and long-wavelength sensitive), and therefore, to visualize a hummingbird's perception of flower colour, flower colour reflectance can be plotted in tetrahedral colour space. Within this space, hue can be calculated as the horizontal, azimuth angle from the positive *X*-axis (*θ*) (Endler and Mielke 2005; Stoddard and Prum 2008). We used visual system data (spectral sensitivities for each of the four cone types) from the hummingbird, *Sephanoides sephanoides* (Herrera *et al*. 2008). Unlike birds, bees have three photoreceptors (UV, short- and medium-wavelength sensitive), and floral reflectance can be plotted in a two-dimensional colour hexagon. In bee colour space, hue is the angle around the origin (Chittka 1992). We used visual system data for the honey floral, *Apis mellifera* (Menzel and Backhaus 1991), for calculating floral hue from the bee's perspective.

We estimated the effect of pathway type on the three measures of hue using phylogenetic analysis of variance (ANOVA) (Garland *et al*. 1993). Unlike standard ANOVA, these methods account for non-independence among data points due to shared evolutionary history (Felsenstein 1985). We used the phylogeny from Ng and Smith (2016) and pruned the tree to only include the 27 study species. In these analyses, hue was treated as the response variable and the pathway (sole anthocyanins, or dual anthocyanins and carotenoids) as the predictor. We applied two models of trait evolution: Brownian motion (Felsenstein 1985), which assumes that the variance–covariance matrix is directly predicted by the branch lengths, and the Ornstein–Uhlenbeck (OU) model, which allows the matrix to be scaled to fit the pattern of trait variation (Martins and Hansen 1997). We also conducted these analyses assuming no phylogenetic effect (equivalent to a standard ANOVA). All ANOVAs were performed using the gls function (nlme package) in R (R Development Core Team 2014; Pinheiro *et al*. 2015). We then compared the fit of each model using Akaike information criterion (AIC) scores, whereby we considered models to be significantly different if the difference between AIC scores (ΔAIC) was greater than two units (Burnham and Anderson 2002).

### Determining the relationship between anthocyanin composition and carotenoid presence

We used similar phylogenetic statistical analyses to test the relationship between the presence of carotenoids and the type of anthocyanin produced. Specifically, we hypothesized that red flowers with floral carotenoids would contain purple or blue (cyanidin- or delphinidin-based, respectively) anthocyanins, while those without the modifying effect of yellow–orange carotenoids would require red pelargonidin-based anthocyanins to achieve the same hue. To investigate this hypothesis, we conducted univariate and multivariate ANOVAs as described in the previous section. In this case, we treated the presence or absence of carotenoids as predictor variables and the relative abundance of the three anthocyanidin types as response variables. We did not include *Schizanthus grahamii* in any of our analyses, as we were unable to score carotenoid presence or absence in this species **[see Supporting Information—Table S1]**. All multivariate analyses of variance (MANOVAs) and ANOVAs were again conducted with the gls function in R with AIC used to assess the fit of each model.

## Results

### Anthocyanidins and carotenoids in red floral tissue

**Figure 2.**
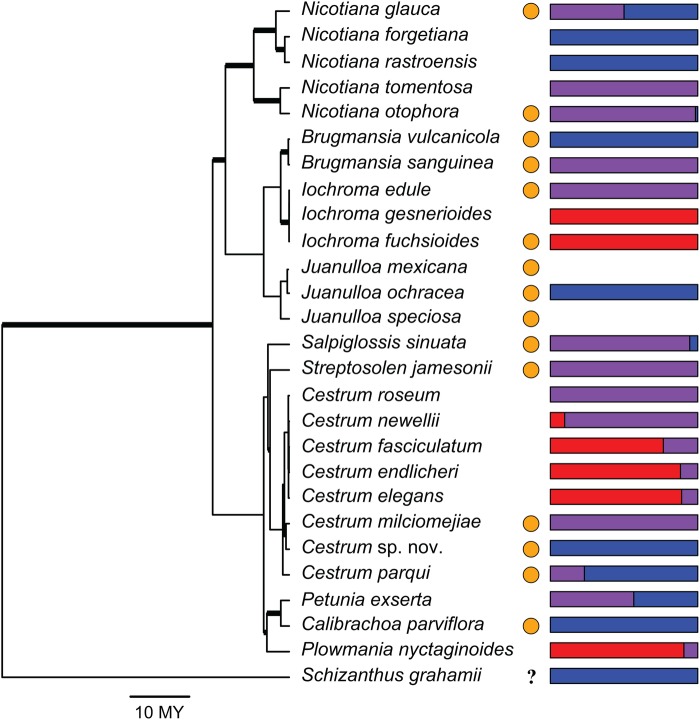
Phylogenetic relationships of red-flowered Solanaceae from a maximum likelihood analysis by Ng and Smith (2016) with all non-red-flowered species pruned from the tree. Bar charts indicate the proportion of pelargonidin (red), cyanidin (purple) and delphinidin (blue) found in the floral tissue, with no bars indicating that the floral tissue had no anthocyanins. Orange circles represent the presence of carotenoids in the floral tissue. Thicker branches of the phylogeny indicate maximum likelihood bootstrap support greater than or equal to 70 %. The scale bar indicates time in millions of years (MY). *Schizanthus grahamii* was included although we were not able to score carotenoid presence/absence for this species (indicated by the question mark).

We found pelargonidin-, cyanidin- and delphinidin-based anthocyanins in red-flowered Solanaceae that included five of the six common anthocyanidins [delphinidin, cyanidin, petunidin, pelargonidin and malvidin; Fig. 2, **see Supporting Information—Table S1**]. Delphinidin-based anthocyanins were most commonly produced, either alone or in combination with cyanidin-based anthocyanins. On the other hand, relatively few species produced pelargonidin-based anthocyanins and, when they did, pelargonidin was rarely produced alone. Instead, pelargonidin was more often found with cyanidin-based anthocyanins. We did not find any flowers that produced pelargonidin- and delphinidin-based anthocyanins together, and two of the red-flowered Solanaceae did not produce any anthocyanins. Our findings are consistent with previous studies that have reported the anthocyanidins in six of the red-flowered Solanaceae (Robinson and Robinson 1934; Beale *et al*. 1941; Miller *et al.* 1967; Ando *et al.* 1999, 2000; Waterworth and Griesbach 2001; Smith and Rausher 2011), with the exception of finding delphinidin to be additionally present in *Calibrachoa parviflora* (Waterworth and Griesbach 2001).

We found that *N. glauca*, which was not sampled in our previous survey, produces carotenoids as well as anthocyanins. This brings the total number of species that produce both anthocyanins and carotenoids to 13 out of 27 species, while 11 solely produce anthocyanins [Fig. 2, **see Supporting Information—Table S1**].

### Hue of flowers using different pathway types

**Figure 3.**
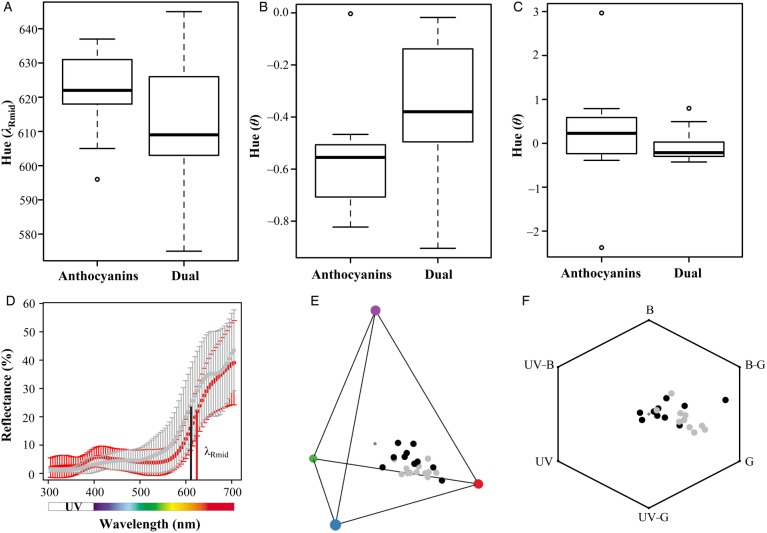
Boxplots showing the hue of red Solanaceae flowers that solely produce anthocyanins and those that dually produce anthocyanins and carotenoids (A–C). Hue is represented by three metrics: *λ*_Rmid_ (A and D), and as perceived by hummingbird (*θ*; B and E) and bee (*θ*; C and F). (D) Reflectance spectra averaged across species that produce anthocyanins (red circles) and those that produce both anthocyanins and carotenoids (grey circles), with red and black lines indicating *λ*_Rmid_ for each spectral curve, respectively. Vertical bars represent standard deviation. Flower colour is also shown in hummingbird tetrahedral space (E) and bee hexagon space (F) with black circles representing anthocyanin-producing flowers and grey circles representing flowers that produce both anthocyanins and carotenoids. The smaller dark grey circle in the colour spaces indicates the achromatic centre. The corners of both colour spaces indicate the excitation of photoreceptors: (E) blue, green, red and purple circles represent short, medium, long and ultraviolet wavelength, respectively; (F) UV, ultraviolet; UV-B, UV-blue; B, blue; B-G, blue-green; G, green; UV-G, UV-green.

We found a general trend that red flowers that produced both carotenoids and anthocyanins had a shorter *λ*_Rmid_ (towards more orange–red hues) than those flowers that solely produced anthocyanins [Fig. 3A and D, **see Supporting Information—Fig. S1**]. This trend of a shift in hue was also found when considering hue under a hummingbird and bee visual model (Fig. 3B, C, E and F). However, for all metrics of hue, the non-phylogenetic ANOVA showed that there was no significant difference in hue between red flowers that solely produced anthocyanins and those that additionally produced carotenoids [*P*> 0.05; **see Supporting Information—Table S2**]. Similarly, the phylogenetic ANOVA conducted using an OU model, which was a better fit than Brownian motion (ΔAIC = 7.88–40.28), also supported the conclusion that the hue of red Solanaceae flowers does not significantly vary with the presence or absence of carotenoids [*P*> 0.05; **see Supporting Information—Table S2**].

### The relationship between anthocyanin composition and carotenoid presence

Multivariate and univariate ANOVAs support a trade-off between the presence of carotenoids and floral anthocyanin composition. Both non-phylogenetic MANOVAs and phylogenetic MANOVAs with the OU model were a better fit than those with a Brownian motion model (ΔAIC = 55.08–56.95) and were indistinguishable from one another (ΔAIC < 2). Both of these models suggested that the presence or absence of floral carotenoids had a significant effect on the type of anthocyanins produced [*P*= 0.02–0.04; **see Supporting Information—Table S3**]. The ANOVAs with individual anthocyanin types suggested that this effect was driven by a difference in the proportion of pelargonidin produced (*P*= 0.02–0.04). These results follow the pattern shown in Fig. 2, where red-flowered species that primarily produce ‘red’ pelargonidin-based pigments typically do not have carotenoid expression (5 of 6 species), while the remaining species, which produce only ‘purple’ cyanidin- or ‘blue’ delphinidin-derived anthocyanins, tend to additionally express carotenoids (12 of 18 species). We found no relationship in the univariate ANOVAs between the proportion of cyanidin or delphinidin produced and the presence of carotenoids (*P*> 0.05). This is not surprising given the wide variation in proportions of these two anthocyanidin classes among the carotenoid-expressing set of taxa (Fig. 2).

## Discussion

Our study demonstrates that red flowers in Solanaceae produce five of the six common anthocyanidins, spanning all three hydroxylation levels. Many of these red-flowered species also express carotenoid pigments, although both pigment pathways for making red flowers (solely anthocyanins or anthocyanins plus carotenoids) produce similar shades of red, even when considering the visual system of pollinators. Behavioural studies would be required to ascertain whether these slight differences in red hue could be discriminated by pollinators. We also found that the red-flowered species without carotenoids tend to produce the ‘red’ monohydroxylated anthocyanins, while species with carotenoids often use the ‘purple’ and ‘blue’ di- and tri-hydroxylated anthocyanins. These patterns suggest two common ways to make red flowers in Solanaceae: the sole production of red anthocyanins or the dual production of purple/blue anthocyanins and orange carotenoids. There are, however, some exceptions to this pattern [e.g. *Nicotiana rastroensis* and *Iochroma fuchsioides*; Fig. 2, **see Supporting Information—Table S1**], pointing to additional factors influencing flower colour. Here, we discuss these findings in light of our current understanding of the evolution and development of red flowers across flowering plants.

### The pigments underlying red flowers

Our results, along with previous studies, suggest that a diversity of pigments and combinations of pigments can make red flowers. Pelargonidin has most commonly been found to underlie red flower colouration, either alone (e.g. *Ipomoea*, Zufall and Rausher 2004; Des Marais and Rausher 2010; *Iochroma*, Smith and Rausher 2011; *Penstemon*, Wessinger and Rausher 2015) or in combination with cyanidin (e.g. *Mimulus*, Pollock *et al*. 1967; *Aquilegia*, Taylor 1984; *Iochroma*, A.E. Berardi, S. Hildreth, B.S.J. Winkel and S.D. Smith, in review). Cyanidin alone has also been reported (e.g. *Phlox*, Hopkins and Rausher 2011), and in some instances, cyanidin-producing flowers are red due to a lower vacuolar pH (e.g. Yoshida *et al*. 2003). In contrast, the use of delphinidin-based anthocyanins in red flowers appears to be rare, with only a few red-flowered species (e.g. *Boronia elatior* and *Lathyrus grandiflorus*) and varieties (e.g. *Linum grandiflorum* and *Hydrangea hortensis*) known to produce delphinidin-based anthocyanins (Robinson and Robinson 1931, 1934; Toki *et al.* 1995).

Our results show that red-flowered Solanaceae differ from other groups by most commonly using delphinidin-based anthocyanins, or a combination of delphinidin- and cyanidin-based anthocyanins to make red flowers. However, with the modifying effect of carotenoids on floral colour, these delphinidin- and cyanidin-producing Solanaceae have converged on the same floral hue as those producing red pelargonidin pigments (Fig. 3). There are some exceptions to this trend (Fig. 2), and in these cases, other factors, such as pH and cell shape, also likely play a role. For example, a lower vacuolar pH has previously been shown to contribute to red flower colouration in *Petunia exserta*, and cultivated varieties of *Petunia* and *Calibrachoa* (Griesbach 1996; Griesbach *et al.* 1999; Waterworth and Griesbach 2001). Overall, our results along with previous studies demonstrate that different anthocyanin pigments have been recruited for red flower production in different angiosperm lineages.

### The mechanisms underlying red flower evolution

Molecular studies thus far suggest that the genetic and biochemical changes responsible for the evolution of flower colour differences may often be predictable (Streisfeld and Rausher 2011; Wessinger and Rausher 2012, 2014). Indeed, evolutionary transitions to red flowers have been shown to be associated with shifts towards less-hydroxylated pigments through the inactivation or down-regulation of the enzymes responsible for producing cyanidin- and delphinidin-based anthocyanins in a wide range of taxa (Des Marais and Rausher 2010; Hopkins and Rausher 2011; Smith and Rausher 2011; Wessinger and Rausher 2012, 2014). However, our finding that a number of Solanaceae lineages make red flowers using delphinidin-based anthocyanins in combination with carotenoid pigments suggests that the addition of carotenoids may be another pathway to the evolution of red flowers, and therefore, the genetic changes underlying shifts to red flowers may not be as predictable as previously suggested.

The prevalence of carotenoids in red flowers underscores the need for an increased understanding of the genetic mechanisms controlling variation in the production of these pigments. Although the carotenoid biosynthetic pathway is deeply conserved and well characterized in a number of plant groups (Ruiz-Sola and Rodríguez-Concepción 2012), relatively little is known about the regulation of the pathway or about the types of mutations that can contribute to flower colour differences. Studies thus far suggest that changes in floral carotenoids are predominantly caused by differential expression of carotenoid biosynthetic genes, which could be due to either *cis-*regulatory mutations at these loci or changes in their transcriptional regulators (Yamagishi *et al*. 2010; Zhu *et al*. 2010; Ohmiya 2013; Sagawa *et al*. 2016). However, in some cases, structural mutations in genes outside of the carotenoid biosynthetic pathway have also been implicated. For example, a loss-of-function mutation in a carotenoid cleavage dioxygenase (CCD) gene can block the breakdown of carotenoids into volatiles and thus cause an increase in floral carotenoid accumulation (e.g. Ohmiya *et al*. 2006; Zhang *et al*. 2015). Also, a gain-of-function mutation at the *Orange/Or* gene has also been shown to influence carotenoid accumulation in cauliflower by controlling the differentiation of chromoplasts from proplastids (Li *et al*. 2001). Together, these cases suggest that floral carotenoid production may evolve either through regulatory changes that alter the expression of carotenoid biosynthetic genes or by structural mutations in genes outside of the pathway. The lack of functional variation in the pathway genes suggests strong constraint on these enzymes, which may relate to the role of carotenoids as accessory pigments for photosynthesis (Hirschberg 2001). However, additional studies will be needed to identify the spectrum of mutations that can alter floral carotenoid production as well as the degree to which any of these genetic mechanisms are predictably involved in evolutionary flower colour transitions.

### The role of pleiotropy in red flower pigment production

Evolutionary shifts to red flowers are widely thought to be an adaptive response to pollinator-mediated selection (Fenster *et al*. 2004; Rodríguez-Gironés and Santamaría 2004; Muchhala *et al*. 2014). However, flower colour variation may also evolve indirectly through selection on non-floral traits, such as leaf or fruit pigmentation (Armbruster 2002). Correlated variation in pigment intensity across plant tissues has been well documented in many species (Warren and Mackenzie 2001), but less is known about correlated variation in pigment type. In the case of red-flowered species, vegetative tissue often appears to produce different types of pigments to those found in flowers (Des Marais and Rausher 2010; Wessinger and Rausher 2014), suggesting independent regulation of pigmentation in these tissues. Nonetheless, the potential for correlated changes in flower and fruit pigmentation is essentially unexplored.

Solanaceae provides an ideal system for future work to dissect the extent of correlated evolution in pigment production across plant tissues and the possible role of pleiotropy in flower colour evolution. In addition to its flower colour diversity (Knapp 2010), Solanaceae shows tremendous variation in fruit colour, including green, red, orange, yellow and purple hues (Knapp 2010; Dhar *et al*. 2015; Wang *et al*. 2015). As in flowers, both anthocyanin and carotenoids contribute to these colour differences in fruits (Galpaz *et al*. 2006; Sadilova *et al*. 2006; Paran and van der Knaap 2007; Lightbourn *et al*. 2008; Zeng *et al*. 2014). If evolutionary transitions in flower colour are correlated with changes in fruit colour, then the genetic architecture of pigmentation in these two tissues may involve pleiotropic loci (Smith 2016). In contrast, if pigmentation in these tissues is controlled by different loci, flower colour and fruit colour may evolve independently and respond to the distinct pressures imposed by pollinators, seed dispersers, predators and other agents of selection (Lomáscolo *et al*. 2010; Stournaras *et al*. 2013).

## Conclusions

Our study reveals that the convergent evolution of red flower colouration in Solanaceae is due to the recruitment of different pigment pathways and different classes of pigments. This pattern suggests that the underlying genetic changes are also likely to be diverse and may depend to some degree on the ancestral state in the clade where red flowers arose (Ng and Smith 2016). For example, if the ancestral state is yellow due to carotenoids, the transition to red flowers would almost certainly require novel expression of floral anthocyanins. In contrast, if the ancestral state is the presence of purple anthocyanins, red flowers could evolve either by novel production of floral carotenoids or by a shift towards the production of less-hydroxylated anthocyanins. Previous studies have provided us with strong candidate genes for changes in floral anthocyanins (reviewed in Wessinger and Rausher 2012), and such mechanistic detail is beginning to emerge for floral carotenoids (Ohmiya *et al*. 2006; Zhang *et al*. 2015; Sagawa *et al*. 2016). By placing biochemical, phenotypic and genomic data into the context of a well-resolved phylogeny, future studies of red-flowered Solanaceae and their relatives can trace the suite of changes required to achieve flower colour transitions and determine the degree to which the mechanisms involved depend on evolutionary history (Marazzi *et al*. 2012).

## Sources of Funding

This work was supported by a National Science Foundation grant to S.D.S. (NSF-DEB 1413855).

## Contributions by the Authors

Both authors contributed to data collection, analyses and manuscript preparation.

## Conflict of Interest Statement

None declared.

## Supporting Information

The following additional information is available in the online version of this article –

**Figure S1.** Plot showing the floral hue of red-flowered Solanaceae species that solely produce anthocyanins and those that dually produce anthocyanins and carotenoids. Within these two groups, species are categorized by the highest produced type of anthocyanin (pelargonidin, cyanidin or delphinidin) and represented as dots.

**Table S1.** Pigment and source information for study species. The relative proportion of each anthocyanidin type and presence of carotenoids in the floral tissue of each species are included, in addition to the spectral hue, the part of the flower from which reflectance data were obtained and whether the species is polymorphic in flower colour.

**Table S2.** Non-phylogenetic and phylogenetic ANOVA results comparing the hue of red Solanaceae flowers that solely produce anthocyanins and those that additionally produce carotenoids. The results using three metrics of hue are shown: reflectance at the midpoint between the minimum and maximum reflectance (*λ*_Rmid_), and metrics that consider how red flowers are perceived by hummingbirds and bees (*θ*).

**Table S3.** Results of non-phylogenetic and phylogenetic ANOVAs and MANOVAs testing the effect of pathway (anthocyanin or both anthocyanin and carotenoid) on the type of anthocyanin produced in red Solanaceae flowers.

Additional Information
