# Supplementary material for: How to make a red flower: the combinatorial effect of pigments
Source: AoB Plants. 2016 Mar 1;8:plw013. doi: 10.1093/aobpla/plw013 (PMC4804202; doi:10.1093/aobpla/plw013)
Supplement: Additional Information [file supp_8_plw013_index.html]

How to make a red flower: the combinatorial effect of pigments — Additional Information 

# How to make a red flower: the combinatorial effect of pigments

## Additional Information

Additional Information

- Supplementary Table 1 - docx file
- Supplementary Table 2 - docx file
- Supplementary Table 3 - docx file
- Supplementary Figure 1 - pdf file
