# Supplementary material for: How to make a red flower: the combinatorial effect of pigments
Source: AoB Plants. 2016 Mar 1;8:plw013. doi: 10.1093/aobpla/plw013 (PMC4804202; doi:10.1093/aobpla/plw013)
Supplement: Additional Information [file supp_plw013_plw013supp_table1.docx]

| **Species** | **Anthocyanidin (proportion)** | | | | | |
| --- | --- | --- | --- | --- | --- | --- |
|  | **Pelargonidin** | **Cyanidin-based** | | **Delphinidin-based** | | |
|  |  | **Cyanidin** | **Peonidin** | **Delphinidn** | **Petunidin** | **Malvidin** |
| *Brugmansia sanguinea* | 0 | 1 | 0 | 0 | 0 | 0 |
| *Brugmansia vulcanicola* | 0 | 0 | 0 | 1 | 0 | 0 |
| *Calibrachoa parviflora* | 0 | 0 | 0 | 0.51 | 0.43 | 0.06 |
| *Cestrum elegans* | 0.89 | 0.11 | 0 | 0 | 0 | 0 |
| *Cestrum endlicheri* | 0.88 | 0.12 | 0 | 0 | 0 | 0 |
| *Cestrum fasciculatum* | 0.77 | 0.23 | 0 | 0 | 0 | 0 |
| *Cestrum milciomejiae* | 0 | 1 | 0 | 0 | 0 | 0 |
| *Cestrum newelli* | 0.10 | 0.90 | 0 | 0 | 0 | 0 |
| *Cestrum parqui* | 0 | 0.23 | 0 | 0.77 | 0 | 0 |
| *Cestrum roseum* | 0 | 1 | 0 | 0 | 0 | 0 |
| *Cestrum sp nov* | 0 | 0 | 0 | 0 | 1 | 0 |
| *Iochroma edule* | 0 | 1 | 0 | 0 | 0 | 0 |
| *Iochroma fuchsioides* | 1 | 0 | 0 | 0 | 0 | 0 |
| *Iochroma gesnerioides* | 1 | 0 | 0 | 0 | 0 | 0 |
| *Juanulloa mexicana* | 0 | 0 | 0 | 0 | 0 | 0 |
| *Juanulloa ochracea* | 0 | 0 | 0 | 1 | 0 | 0 |
| *Juanulloa speciosa* | 0 | 0 | 0 | 0 | 0 | 0 |
| *Nicotiana forgetiana* | 0 | 0 | 0 | 1 | 0 | 0 |
| *Nicotiana glauca* | 0 | 0.5 | 0 | 0.5 | 0 | 0 |
| *Nicotiana otophora* | 0 | 0.98 | 0 | 0.02 | 0 | 0 |
| *Nicotiana rastroensis* | 0 | 0 | 0 | 1 | 0 | 0 |
| *Nicotiana tomentosa* | 0 | 1 | 0 | 0 | 0 | 0 |
| *Petunia exserta* | 0 | 0.57 | 0 | 0.43 | 0 | 0 |
| *Plowmania nyctaginoides* | 0.91 | 0.09 | 0 | 0 | 0 | 0 |
| *Salpiglossis sinuata* | 0 | 0.95 | 0 | 0.05 | 0 | 0 |
| *Schizanthus grahamii* | 0 | 0 | 0 | 0.17 | 0.83 | 0 |
| *Streptosolen jamesonii* | 0 | 1 | 0 | 0 | 0 | 0 |

| Species | Carotenoids | Polymorphic | Flower part measured | λRmid |
| --- | --- | --- | --- | --- |
|  |  | in color | for reflectance |  |
| *Brugmansia sanguinea* | Y | N | Lobe | 606 |
| *Brugmansia vulcanicola* | Y | N | - | - |
| *Calibrachoa parviflora* | Y | Y | Lobe | 626 |
| *Cestrum elegans* | N | N | Midpoint tube exterior | 605 |
| *Cestrum endlicheri* | N | N | Midpoint tube exterior | 631 |
| *Cestrum fasciculatum* | N | N | Midpoint tube exterior | 626 |
| *Cestrum milciomejiae* | Y | N | Midpoint tube exterior | 630 |
| *Cestrum newelli* | N | N | Midpoint tube exterior | 618 |
| *Cestrum parqui* | Y | Y | Midpoint tube exterior | 645 |
| *Cestrum roseum* | N | N | - | - |
| *Cestrum sp nov* | Y | N | - | - |
| *Iochroma edule* | Y | Y | Midpoint tube exterior | 606 |
| *Iochroma fuchsioides* | Y | N | Midpoint tube exterior | 604 |
| *Iochroma gesnerioides* | N | N | Midpoint tube exterior | 619 |
| *Juanulloa mexicana* | Y | Y | Upper tube exterior | 575 |
| *Juanulloa ochracea* | Y | Y | Midpoint tube exterior | 575 |
| *Juanulloa speciosa* | Y | Y | Upper tube exterior | 582 |
| *Nicotiana forgetiana* | N | N | Lobe | 637 |
| *Nicotiana glauca* | Y | Y | - | - |
| *Nicotiana otophora* | Y | Y | Midpoint tube exterior | 612 |
| *Nicotiana rastroensis* | N | Y | Lobe | 623 |
| *Nicotiana tomentosa* | N | Y | Lobe | 637 |
| *Petunia exserta* | N | N | Lobe | 621 |
| *Plowmania nyctaginoides* | N | N | Lobe | 596 |
| *Salpiglossis sinuata* | Y | Y | Lobe | 622 |
| *Schizanthus grahamii* | no data | Y | - | - |
| *Streptosolen jamesonii* | Y | Y | Lobe | 603 |

| **Species** | **Source** | **Voucher/Accession number** |
| --- | --- | --- |
| *Brugmansia sanguinea* | Field: Botijlaca, Bolivia | Smith *s.n*. |
| *Brugmansia vulcanicola* | Field: Totoro, Colombia | Ng 026 (COLO) |
| *Calibrachoa parviflora* | Greenhouse | Home Depot |
| *Cestrum elegans* | Field: Banderilla, Mexico | Ng 008 (COLO) |
| *Cestrum endlicheri* | Field: Chiconquaico, Mexico | Ng 010 (COLO) |
| *Cestrum fasciculatum* | Field: Naolinco de Victoria, Mexico | Ng 009 (COLO) |
| *Cestrum milciomejiae* | Field: Presa de Valdesia, Dominican Republic | Montero-Castro et al. 291 (NY) |
| *Cestrum newelli* | Field: Talquian, Mexico | Ng 012 (COLO) |
| *Cestrum parqui* | Field: Icla, Bolivia | Nee 60895 (LPB) |
| *Cestrum roseum* | Cultivated: Botanical Gardens, Bogotá, Colombia | Ng 029 (COL) |
| *Cestrum sp nov* | Herbarium | Jimenez et al. 2893 (JBSD) |
| *Iochroma edule* | Greenhouse | Smith 259 (WIS) |
| *Iochroma fuchsioides* | Greenhouse | Smith 219 (WIS) |
| *Iochroma gesnerioides* | Greenhouse | Nijmegen Accn. #934750129 |
| *Juanulloa mexicana* | Field: Cacahoatan, Mexico | Ng 019 (COLO) |
| *Juanulloa ochracea* | Field: Florencia, Colombia | Ng 022 (COL) |
| *Juanulloa speciosa* | Field: Ibague, Colombia | Ng 023 (COL) |
| *Nicotiana forgetiana* | Greenhouse | USDA National Plant Germplasm System Accn. #555501 |
| *Nicotiana glauca* | Herbarium | Nee et al. 51725 (NY); Nee & Flores 54883 (NY) |
| *Nicotiana otophora* | Cultivated: Jardin Botanico Martin Cardenas, Cochabamba, Bolivia | - |
| *Nicotiana rastroensis* | Greenhouse | Holtsford, *s.n.* |
| *Nicotiana tomentosa* | Field: Torrebamba near Lanturachi, Peru; dried flower tissue used | Smith 548 (HOXA) |
| *Petunia exserta* | Greenhouse (Commercial) | Annie's Annuals ID#3809 (www.anniesannuals.com) |
| *Plowmania nyctaginoides* | Field: Talquian, Mexico | Ng 013 (COLO) |
| *Salpiglossis sinuata* | Greenhouse (Commercial) | Johnny Seeds ID#1390 (www.johnnyseeds.com) |
| *Schizanthus grahamii* | Herbarium | Ferrucci et al. 3022 (NY) |
| *Streptosolen jamesonii* | Greenhouse (Commercial) | Annie's Annuals ID#1008 (www.anniesannuals.com) |
