# Supplementary material for: How to make a red flower: the combinatorial effect of pigments
Source: AoB Plants. 2016 Mar 1;8:plw013. doi: 10.1093/aobpla/plw013 (PMC4804202; doi:10.1093/aobpla/plw013)
Supplement: Additional Information [file supp_plw013_plw013supp_table2.docx]

| **Table S2.** |  |  |  |  |  |  |
| --- | --- | --- | --- | --- | --- | --- |
|  | **Non-phylogenetic** | | **Phylogenetic (Brownian)** | | **Phylogenetic (OU)** | |
| **Hue** | AIC | *p*-value | AIC | *p*-value | AIC | *p*-value |
| λ*_R_*_mid_ | 166.5894 | 0.1799 | 200.9752 | < 0.0001 | 168.5431 | 0.1556 |
| Hummingbird visual system (θ) | 11.29831 | 0.1305 | 53.54808 | 0.0012 | 13.26435 | 0.1198 |
| Bee visual system (θ) | 60.67898 | 0.5565 | 64.28441 | 0.5256 | 56.40516 | 0.7917 |
