# Supplementary material for: How to make a red flower: the combinatorial effect of pigments
Source: AoB Plants. 2016 Mar 1;8:plw013. doi: 10.1093/aobpla/plw013 (PMC4804202; doi:10.1093/aobpla/plw013)
Supplement: Additional Information [file supp_plw013_plw013supp_table3.docx]

| **Table S3.** |  |  |  |  |  |  |
| --- | --- | --- | --- | --- | --- | --- |
|  | **Non-phylogenetic** | | **Phylogenetic (Brownian)** | | **Phylogenetic (OU)** | |
| **ANOVA** | AIC | *p*-value | AIC | *p*-value | AIC | *p*-value |
| Pelargonidin ~ Pathway | 29.83365 | 0.038 | 86.78543 | 0.0001 | 31.70365 | 0.0216 |
| Cyanidin ~ Pathway | 38.51635 | 0.4373 | 90.20427 | 0.0004 | 49.43027 | 0.4439 |
| Delphinidin ~ Pathway | 37.49162 | 0.3071 | 47.25098 | 0.858 | 32.58917 | 0.832 |
| **MANOVA** |  |  |  |  |  |  |
| Pelargonidin+Cyanidin+Delphinidin ~ Pathway | 29.83365 | 0.038 | 86.78543 | 0.0001 | 31.70365 | 0.0216 |
