# Supplementary figures and images for: How to make a red flower: the combinatorial effect of pigments
Source: AoB Plants. 2016 Mar 1;8:plw013. doi: 10.1093/aobpla/plw013 (PMC4804202; doi:10.1093/aobpla/plw013)

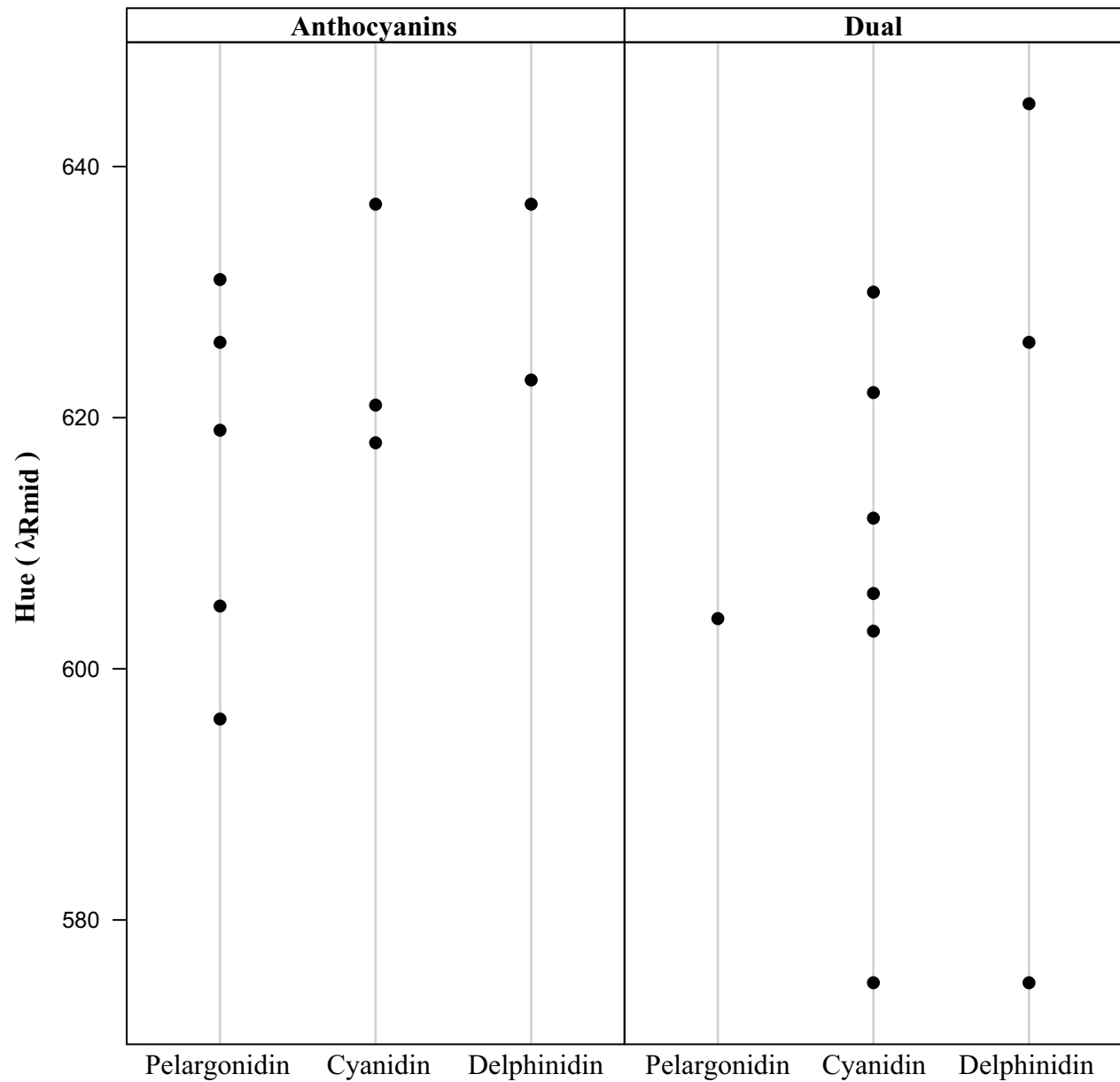

Supplement: Additional Information [file supp_plw013_plw013supp_fig1.pdf]
